# Supplementary material for: Assessing the Various Antagonistic Mechanisms of Trichoderma Strains against the Brown Root Rot Pathogen Pyrrhoderma noxium Infecting Heritage Fig Trees
Source: J Fungi (Basel). 2022 Oct 19;8(10):1105. doi: 10.3390/jof8101105 (PMC9605450; doi:10.3390/jof8101105)
Supplement: Supplementary file 1 [file jof-08-01105-s001.zip › jof-1855757-supplementary.pdf]

Table S1. Primer pairs used for gene expression analysis by qPCR technique.

Gene	Transcript ID *Trichoderma* strain #5001	Transcript ID *Trichoderma* strain #5029	Size (bp)	Forward primer (5′-3′)	Reverse primer (5′-3′)
bgn	4761	6905	137	AACAAGCTCAACCATGCGTA	GGCATGTCATCCTTGTTGTT
act	7320	562	89	CTGGCACCACACCTTCTACA	TGGACTTGGGGTTGATGG
chit	4269	7447	103	GGAACCCAAACTCGAAACAA	TCGTCGTCATCGCAATAAAA
endo	7128	4923	136	CCTGCAGATTTGGTCACGTC	CATCGTTCCAGGAATCATCA
qid	3183	107	83	GCTCCTTTGCTACCTGGAAC	CGACTTGGTCTTGGAGTCGT
prb	8501	5207	112	GCCTCCGTTGATGCAGATTCCCAT	TCCAAGTGTCGATGTTGTACG
tef	509	1382	103	TGAGAAGTTCGAGAAGGAAGC	GATGGTGATACCACGCTCAC
pra	5419	2538	101	GTCTACCCTGCCTCCCAGAT	TAGGACGGGTTCACGATGAT

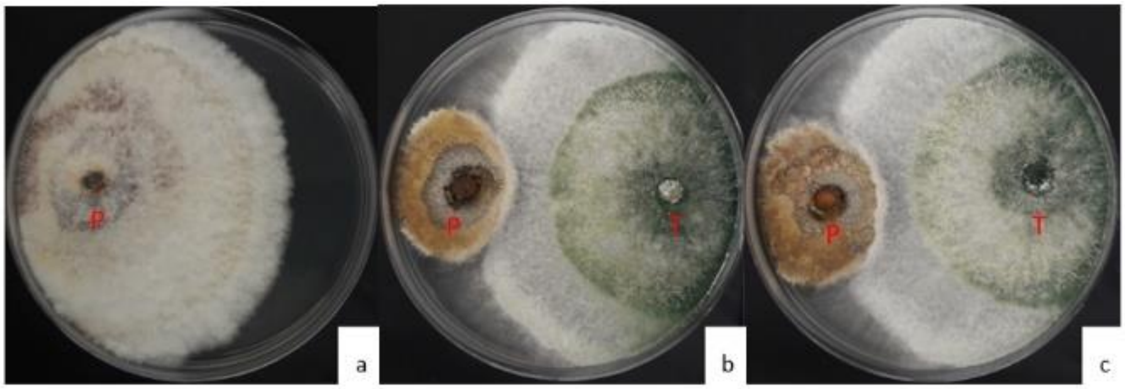

Figure S1. Growth inhibitory effect of the b) *Trichoderma* strain #5001, c) strain #5029 on *P. noxium* strain B after 5 days of inoculation in dual culture (T: *Trichoderma* and P: *P. noxium*), a) *P. noxium* strain B grown alone.

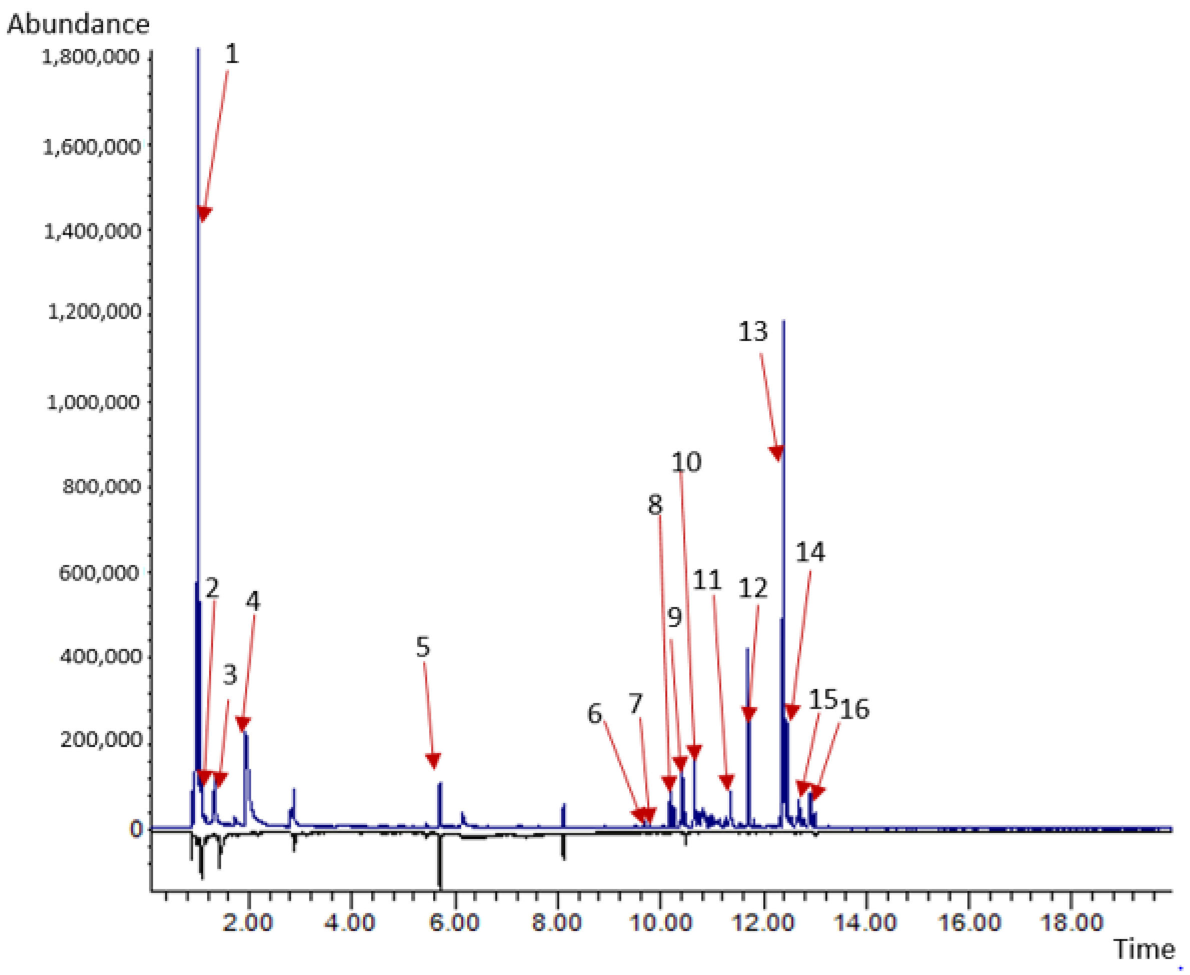

Figure S2. Chromatogram of volatile compounds produced by *P. noxium* strain B (above zero level); chromatogram of volatile compounds detected in the PDA media control (below zero level). Numbers refer to compounds listed in Table S2

Table S2. Volatile compounds produced by *P. noxium* strain B tentatively identified through SPEM GC/MS analysis

Peak number	Compounds	Class	Retention time	Area %
1	Ethanol	Alcohol	1.02	28.47
2	Furan	Furans	1.06	2.88
3	Isobutanol	Alcohol	1.32	2.47
4	Isopentanol	Alcohol	1.94	11.51
5	limonene	monoterpene	6.14	1.01
5	β- necoclovene	Sesquiterpene	9.69	0.31
6	Aristolene	Sesquiterpene	9.78	0.21
7	Thujopsene	Sesquiterpene	10.18	1.61

8	2-isopropenyl-4a,8, dimethyl-1,2,3,4a,5,6,7 octahydronaphthalene	Sesquiterpene	10.42	2.55
9	δ-gurjunene	Sesquiterpene	10.65	3.35
10	β-elemene	Sesquiterpene	11.36	1.34
11	β–caryophyllene	Sesquiterpene	11.70	8.14
12	8-isopropenyl-1,5-dimethyl-cyclodeca-1,5-diene	Sesquiterpene	12.39	22.93
13	unidentified sesquiterpene	Sesquiterpene	12.45	5.27
14	Cadinene	Sesquiterpene	12.68	1.29
15	Elixene	Sesquiterpene	12.91	1.83

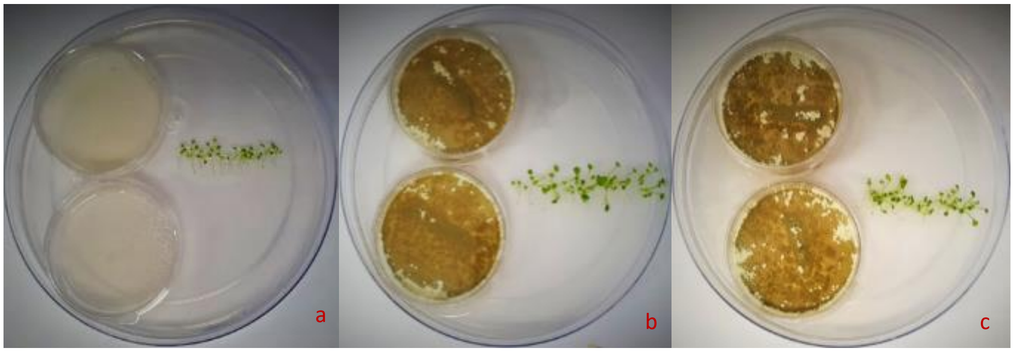

Figure S3. Effect of volatile compounds on the growth of *Arabidopsis thaliana* in a shared atmosphere with *Trichoderma* strains #5001 and #5029 for 12 days. (a) Control plants exposed to PDA medium, (b) plants exposed to *Trichoderma* strain #5001 and (c) plants exposed to the strain #5029.
